# Care of the Terminally Ill Cancer Patient: A Handbook for the Medical Oncologist

**DOI:** 10.1038/sj.bjc.6601649

**Published:** 2004-05-26

**Authors:** M Fallon

**Affiliations:** 1 Edinburgh Cancer Center, UK

I found this book very interesting and refreshing. It has added value as a palliative care text because it is written by an oncologist. Michael Owens covers the complete range of issues that the physician may face when caring for a patient with cancer. By definition some of these are only dealt with briefly; however, all are mentioned in a way that is clinically meaningful. Some of the nonphysical problems, for example socioeconomic, are discussed with a strong North American slant but even this area is not without relevance in Europe.

This text could be very useful for the oncologist in training and even thought provoking for the established oncologist.

